# Optimal Course of Statins for Patients With Aneurysmal Subarachnoid Hemorrhage: Is Longer Treatment Better? A Meta-Analysis of Randomized Controlled Trials

**DOI:** 10.3389/fnins.2021.757505

**Published:** 2021-10-25

**Authors:** Tao Liu, Shiyu Zhong, Qingqing Zhai, Xudong Zhang, Huiquan Jing, Kunhang Li, Shengyu Liu, Shuo Han, Lishuai Li, Xin Shi, Yijun Bao

**Affiliations:** ^1^Department of Neurosurgery, The Fourth Affiliated Hospital of China Medical University, Shenyang, China; ^2^School of Management, Shanghai University, Shanghai, China; ^3^School of Public Health, Capital Medical University, Beijing, China; ^4^School of Maths and Information Science, Shandong Institute of Business and Technology, Yantai, China; ^5^Business School, Manchester Metropolitan University, Manchester, United Kingdom

**Keywords:** statins, aneurysmal, subarachnoid hemorrhage, outcome, complications, meta-analysis

## Abstract

Statins are used in clinical practice to prevent from complications such as cerebral vasospasm (CVS) after aneurysmal subarachnoid hemorrhage (aSAH). However, the efficacy and safety of statins are still controversial due to insufficient evidence from randomized controlled trials and inconsistent results of the existing studies. This meta-analysis aimed to systematically review the latest evidence on the time window and complications of statins in aSAH. The randomized controlled trials in the databases of The Cochrane Library, PubMed, Web of Science, Embase, CNKI, and Wanfang from January 2005 to April 2021 were searched and analyzed systematically. Data analysis was performed using Stata version 16.0. The fixed-effects model (M-H method) with effect size risk ratio (RR) was used for subgroups with homogeneity, and the random-effects model (D-L method) with effect size odds ratio (OR) was used for subgroups with heterogeneity. The primary outcomes were poor neurological prognosis and all-cause mortality, and the secondary outcomes were cerebral vasospasm (CVS) and statin-related complications. This study was registered with PROSPERO (International Prospective Register of Systematic Reviews; CRD42021247376). Nine studies comprising 1,464 patients were included. The Jadad score of the patients was 5–7. Meta-analysis showed that poor neurological prognosis was reduced in patients who took oral statins for 14 days (RR, 0.73 [0.55–0.97]; *I*^2^ = 0%). Surprisingly, the continuous use of statins for 21 days had no significant effect on neurological prognosis (RR, 1.04 [0.89–1.23]; *I*^2^ = 17%). Statins reduced CVS (OR, 0.51 [0.36–0.71]; *I*^2^ = 0%) but increased bacteremia (OR, 1.38 [1.01–1.89]; *I*^2^ = 0%). In conclusion, a short treatment course of statins over 2 weeks may improve neurological prognosis. Statins were associated with reduced CVS. Based on the pathophysiological characteristics of CVS and the evaluation of prognosis, 2 weeks could be the optimal time window for statin treatment in aSAH, although bacteremia may increase.

## Introduction

The annual rate of aneurysmal subarachnoid hemorrhage (aSAH) is 10/100,000 person-years, accounting for Keywords: Statins, Aneurysmal, Subarachnoid Hemorrhage, Outcome, complications, Meta-analysis 4–5% of all strokes, with high mortality and disability rates (Andersen et al., 2019). The incidence of aSAH accounts for ~80% of the cases of non-traumatic subarachnoid hemorrhage (which also includes arteriovenous malformation etc.) (D'Souza, 2015). Andersen et al. (2019) mentioned that patients who suffered from aSAH had a 1-year mortality of 50%, and nearly half of the surviving patients lived with cognitive and functional limitations. Even with early aneurysm clipping or interventional therapy, the 1-year mortality with a Glasgow Coma Scale (GCS) of 3–5 was 65.8% (Lashkarivand et al., 2020). In China, the cumulative 12-month mortality with aSAH was reported to be 24.6%, and aSAH accounted for ~77.4% of all causes of SAH (Bian et al., 2012). Therefore, it is crucial for neurologists to employ a safe and reliable pharmacological therapy for patients with aSAH.

The American Heart Association/American Stroke Association guidelines regarding 3-hydroxy-3-methylglutaryl-coenzyme A reductase inhibitors (statins) for aSAH are inconclusive, and potential values of statins in aSAH should still be explored (Connolly et al., 2012; Juif et al., 2020). The results of many trials are still controversial owing to the insufficient evidence from randomized controlled trials (RCTs) and the inconsistent results of the existing studies (Liu et al., 2013; Lei et al., 2014; Lizza et al., 2014; Sikora Newsome et al., 2015; Akhigbe et al., 2017; Shen et al., 2017). In addition, some complications have been reported in patients with aSAH during statin therapy, such as bacteremia, epilepsy, elevated transaminase, and rhabdomyolysis (Magulick et al., 2014; Guo et al., 2015; Sikora Newsome et al., 2015; Berent et al., 2019; Nikolic et al., 2020). These complications might affect outcomes in patients with aSAH treated with statins. In previous studies, the usual treatment duration was 2 or 3 weeks. Statins are believed to relieve cerebral vasospasm (CVS) (Siasios et al., 2013; Lizza et al., 2014; Sikora Newsome et al., 2015). The high-risk period of CVS is 3–14 days after aSAH, with a peak at 6–8 days, and symptomatic CVS usually disappears after 12 days of aSAH (De Backer, 2016; Berent et al., 2019). Therefore, 2 weeks could be a more reasonable treatment duration. Following a literature update, the RCTs of statins were selected for systematic review and meta-analysis, including recently published trials on the efficacy and safety of statins in aSAH.

In this study, a systematic review and meta-analysis of RCTs of statins for treating aSAH was performed, and the efficacy and safety of statins treatment were evaluated. Based on the pathological characteristics of CVS and possible complications, we hypothesized that a short-course administration of statins might be more reasonable than a longer course.

## Methods

### Search Strategy

We carried out a systematic literature search of articles comparing statins with placebo in patients with aSAH using the keywords [“Statin” and “aneurysmal subarachnoid hemorrhage”] and their synonyms from January 2005 to April 2021 through The Cochrane Library, PubMed, Web of Science, Embase, China National Knowledge Internet (CNKI), and Wanfang. The records were systematically evaluated using the inclusion and exclusion criteria (see below). In the initial search, the discrepancies were resolved by two researchers (TL and SZ). The search strategy is presented in Supplementary Table 1.

**Figure 1:**
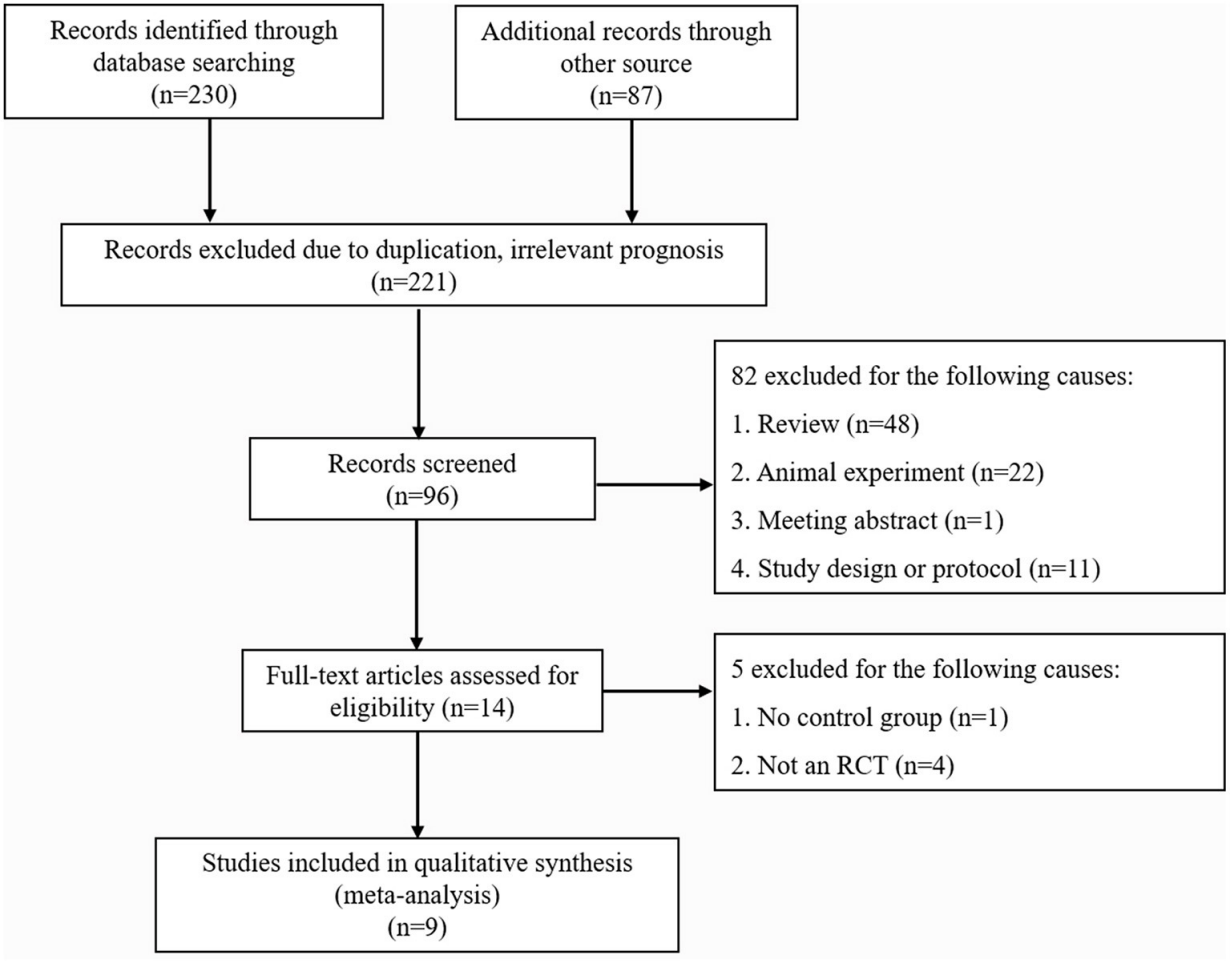
Literature search and selection.

A PRISMA (Preferred Reporting Items for Systematic Reviews and Meta-Analyses) flowchart of the literature search strategy is presented in Figure 1 (Moher et al., 2009). This study was registered with PROSPERO (CRD42021247376).

### Inclusion and Exclusion Criteria

The selected studies met the following inclusion criteria: the study type was RCT; the study subjects met diagnostic criteria for aSAH; articles in Chinese or English; statins used as treatment modality and compared with placebo. Exclusion criteria were duplicate publication, animal studies, unavailable full text, and no clear outcome or efficacy evaluation criteria.

### Data Extraction

Two independent researchers (YB and XS) performed the data extraction and quality assessment using a standardized extraction method, including authors, study design, intervention duration, year of publication, sample size, sex, outcome, and follow-up time.

The primary outcomes were poor neurological prognosis and all-cause mortality. Poor neurological prognosis was defined as a modified Rankin scale (mRS) score of 3–6 or a Glasgow Outcome Score (GOS) of 1–3 (Jennett and Bond, 1975; van Swieten et al., 1988). The secondary outcomes were CVS and statin-related complications, such as bacteremia, epilepsy, elevated transaminase, and rhabdomyolysis.

### Statistical Analysis

Stata 16.0 (StataCorp LP) and RevMan version 5.3 software (Cochrane Collaboration) were used for literature analysis. An inconsistent index (*I*^2^) test, which ranges from 0 to 100%, was used to assess homogeneity among the studies, and both *P* ≥ 0.1 and *I*^2^ ≤ 50% were considered to indicate no heterogeneity.

If the studies were homogeneous, the fixed-effects model (M-H method) with the effect size risk ratio (RR, 95% CI) was used. If the homogeneity condition was not met, a funnel plot, Labbé plot, and sensitivity analysis were used to determine the heterogeneity. Moreover, relevant literature with an excessive impact on model stability was excluded and re-analyzed. If a significant heterogeneity was observed among the studies without distinct reasons, the statistical analysis was performed using the random-effects model (D-L method) with the effect size odds ratio (OR, 95% CI). The chi-square test was used to analyze the results of each study, and differences were considered statistically significant when *P* ≤ 0.05.

## Results

According to the retrieval strategy, 230 articles in English and 87 in Chinese were collected from the databases (Figure 1). After eliminating duplicate articles, those with irrelevant prognosis indicators, reviews, meeting abstracts, and articles describing animal experiments, 14 articles remained. After assessing the 14 full texts for eligibility, we excluded five publications because of lack of controls (one paper) or because they were not RCT studies (four papers). Finally, nine articles were included (Lynch et al., 2005; Tseng et al., 2006; Chou et al., 2008; Vergouwen et al., 2009; Garg et al., 2013; Kirkpatrick et al., 2014; Diringer et al., 2016; Naraoka et al., 2018; Chen et al., 2020). The baseline characteristics of the included trials are summarized in Table 1.

**Table 1:** Characteristics of studies included in the systematic review.

**References**	**Study type**	**Placebo/statins**	**No. of patients**	**Mean Age (years ± SD)**	**Male (%)**	**Course of treatment (days)**	**Follow up time (months)**
Lynch et al. (2005)	RCT	Placebo Simvastatin	20 19	47 ± 8.9 65 ± 18.5	3 (15) 3 (16)	14	NR
Tseng et al. (2006)	RCT	Placebo Pravastatin	40 40	52.0 ± 13.2 53.8 ± 10.7	21 (52.5) 15 (37.5)	14	NR
Chou et al. (2008)	RCT	Placebo Simvastatin	20 19	56 ± 15 50 ± 14	4 (20) 6 (32)	21	NR
Vergouwen et al. (2009)	RCT	Placebo Simvastatin	16 16	54 ± 11 53 ± 11	4 (15) 8 (50)	14	6
Garg et al. (2013)	RCT	Placebo Simvastatin	19 19	48.8 ± 2.4 49.4 ± 1.8	10 (52.6) 11 (57.9)	14	6
Kirkpatrick et al. (2014)	RCT	Placebo Simvastatin	412 391	49 ± 9.8 51 ± 9.5	131 (34) 121 (29)	21	6
Diringer et al. (2016)	RCT	Placebo Simvastatin	12 13	60 ± 10 59 ± 12	4 (33) 5 (38)	21	6
Naraoka et al. (2018)	RCT	Placebo Pitavastatin	54 54	58 ± 12 55 ± 12	20 (37) 14 (26)	14	3
Chen et al. (2020)	RCT	Placebo Atorvastatin	150 150	75.21 ± 1.7 76.1 ± 11.1	63 (42) 73 (48.7)	14	6

*RCT, randomized controlled trials; SD, standard deviation; NR, not reported*.

### Primary Outcomes

**Figure 2:**
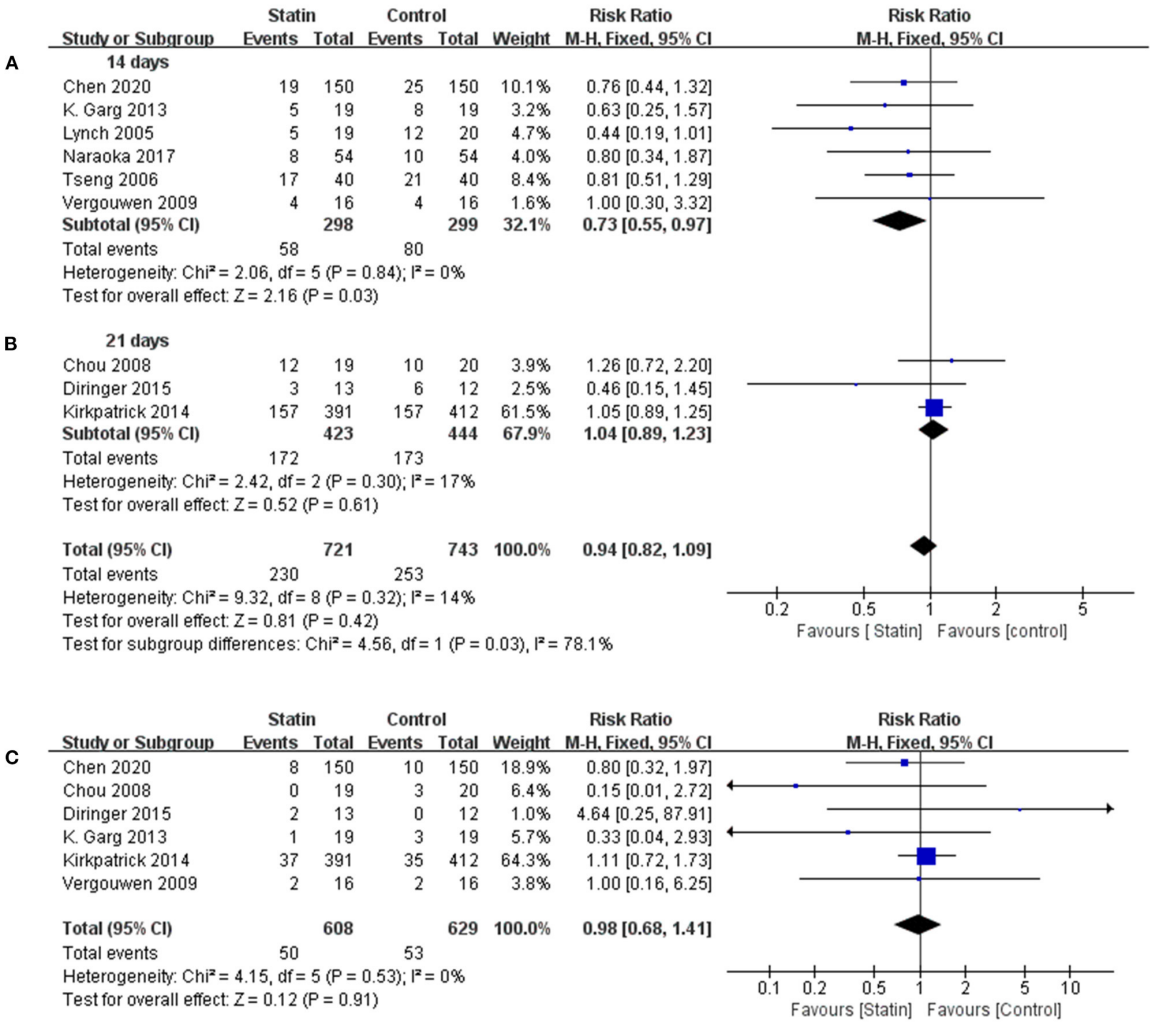
Forest plot comparing the poor neurological prognosis for the course of 14 **(A)** and 21 days **(B)** respectively and all-cause mortality **(C)** between statin and placebo groups.

Considering the two different treatment courses (14 and 21 days), a subgroup analysis was conducted (Figure 2). Since the heterogeneity was not significant in either group (*I*^2^ = 0.0%, *P* = 0.84; *I*^2^ = 17%, *P* = 0.30), a fixed-effects model (M-H method) was used for the analysis. The results showed that taking statins continuously for 14 days reduced the incidence of poor neurological prognosis (RR, 0.73 [0.55–0.97]; *I*^2^ = 0%; Figure 2A). The treatment course of 21 days showed no statistically significant effect on neurological prognosis (RR, 1.04 [0.89–1.23]; *I*^2^ = 17%; Figure 2B). All-cause mortality was evaluated in six RCTs (Chou et al., 2008; Vergouwen et al., 2009; Garg et al., 2013; Kirkpatrick et al., 2014; Diringer et al., 2016; Chen et al., 2020). Pooled analysis found that statins had no effect on mortality (RR, 0.98 [0.68–1.41]; *I*^2^ = 0%; Figure 2C).

### Secondary Outcomes

**Figure 3:**
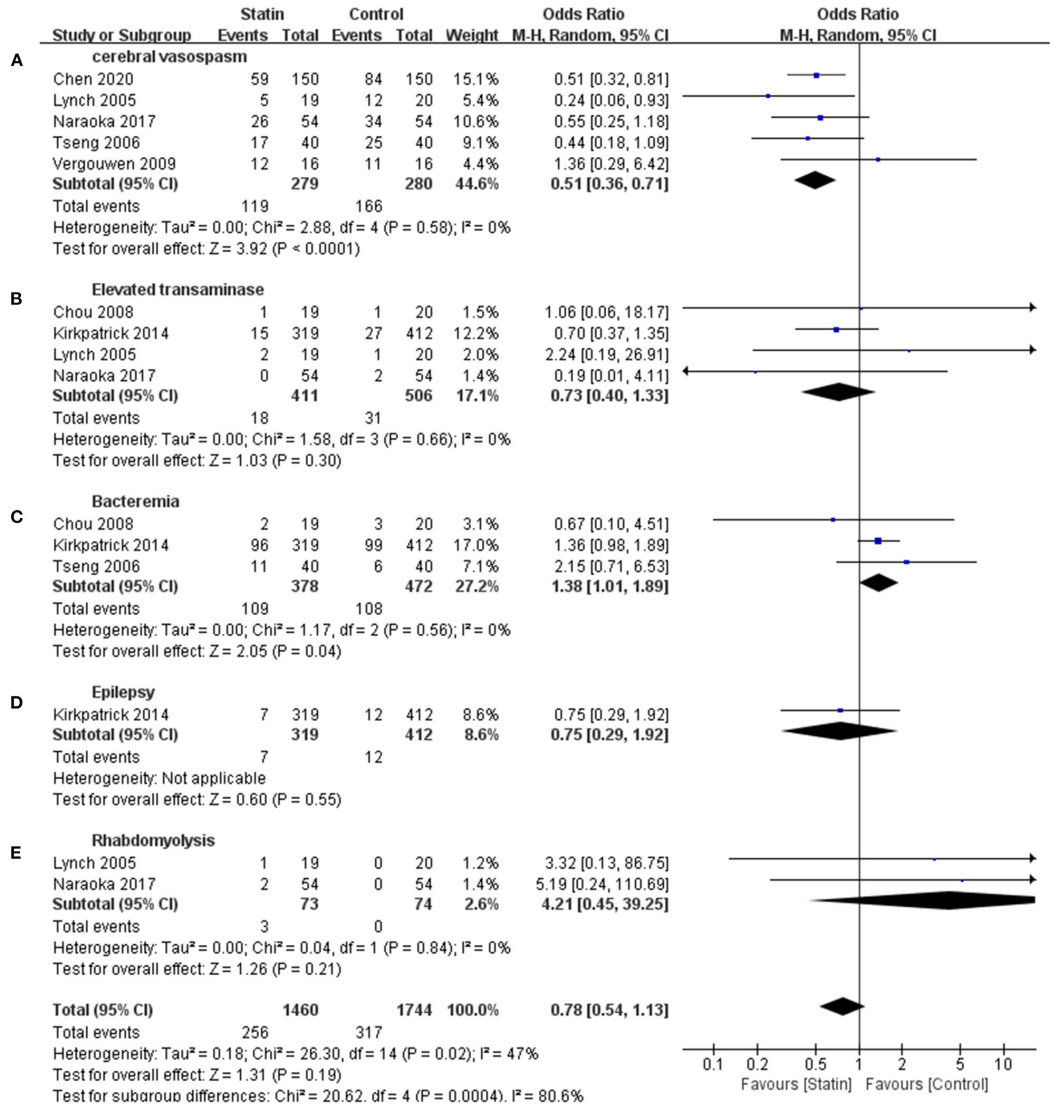
Meta-analysis of complications for statin vs. placebo. **(A)** Lower cerebral vasospasm rate in statin group was demonstrated. **(C)** Increased bacteremia was shown in statin group. **(B,D,E)** Complications events including elevated transaminase, rhabdomyolysis, and epilepsy were similar in both groups.

Seven publications reported CVS and statin-related complications, such as elevated transaminase, bacteremia, rhabdomyolysis, and epilepsy (Lynch et al., 2005; Tseng et al., 2006; Chou et al., 2008; Vergouwen et al., 2009; Kirkpatrick et al., 2014; Naraoka et al., 2018; Chen et al., 2020). This meta-analysis indicated that complications were not significantly different between the statin and control groups (OR, 0.78 [0.54–1.13]; *I*^2^ = 47%; Figure 3), but there was a relatively high heterogeneity. Subgroup analysis revealed a statistically significant difference in the rate of CVS events, suggesting that statins might prevent the occurrence of CVS (OR, 0.51 [0.36–0.71]; *I*^2^ = 0%; Figure 3A). Statins were associated with increased bacteremia (OR, 1.38 [1.01–1.89]; *I*^2^ = 0%; Figure 3C). The risks for elevated transaminase (OR, 0.73 [0.40, 1.13]; *I*^2^ = 0%; Figure 3B), epilepsy (OR, 0.75 [0.29, 1.92]; Figure 3D), and rhabdomyolysis (OR, 4.21 [0.45–39.25]; *I*^2^ = 0%; Figure 3E) were similar in both the statin and placebo groups.

### Quality and Publication Bias of the Included Studies

We used the Jadad score to quantitatively assess the quality of the included studies. Nine studies had a Jadad score of 5–7 points, indicating high quality (Table 2). Regression-based Harbord's and Egger's tests were not statistically significant for either primary or secondary outcomes (Table 3).

**Table 2:** Quality assessment of the included studies.

**References**	**Randomization^a^**	**Concealment of allocation^b^**	**Double blinding^c^**	**Withdrawals and dropouts^d^**	**Jadad score**
Lynch et al. (2005)	1	1	2	1	5
Tseng et al. (2006)	1	1	2	1	5
Chou et al. (2008)	1	1	2	1	5
Vergouwen et al. (2009)	2	2	2	1	7
Garg et al. (2013)	2	0	2	1	5
Kirkpatrick et al. (2014)	2	2	2	1	7
Diringer et al. (2016)	2	0	2	1	5
Naraoka et al. (2018)	1	1	2	1	5
Chen et al. (2020)	1	2	2	1	6

a *Randomization: the method of randomization was described appropriately (2 points); the randomized trials did not describe the methods of random assignment (1 point); not randomized or inappropriate method of randomization (0 points)*.

b *Conealment of allocation: the method of concealment of allocation was described appropriately (2 points); the study only indicated the use of a random number table or other random assignment methods (1 point), not preventing the predictability of grouping (0 points)*.

c *Double-blinding: the randomization method was described appropriately (2 points); the study only stated double-blinding, but the methods were not described (1 point); not double-blind or inappropriate blinding (0 points)*.

d *Withdrawals and dropouts: a description of the numbers and reasons for withdrawals and dropouts (1 point); not follow-up (0 points)*.

*The total Jadad score of 1–3 was considered low-quality studies, and the score of 4–7 was considered high-quality studies*.

**Table 3:** Meta-analysis of various outcomes and publication bias.

**Outcome**	**RR/OR (95%CI)**	**Model**	**Heterogeneity**		***P***	***P*** **-value for publication bias**		**Studies (N)**
			***P***	***I ^**2**^*(%)**		**Harbord's test**	**Egger's test**	
Poor prognosis (14 days)	0.73 (0.55, 0.97)	Fixed	0.84	0	0.03	0.863	0.714	6
Poor prognosis (21 days)	1.04 (0.89, 1.23)	Fixed	0.30	17	0.61	0.138	0.163	3
Mortality	0.98 (0.68, 1.41)	Fixed	0.53	0	0.91	0.228	0.240	6
CVS	0.51 (0.36, 0.71)	Random	0.58	0	0.0001	0.791	0.733	5
Bacteremia	1.38 (1.02, 1.89)	Random	0.56	0	0.04	0.976	0.996	3
Epilepsy	0.75 (0.29, 1.92)	Random	NA	NA	0.55	NA	NA	1
Elevated transaminase	0.73 (0.40, 1.33)	Random	0.66	0	0.30	0.954	0.897	4
Rhabdomyolysis	4.21 (0.45, 39.25)	Random	0.84	0	0.21	0.983	0.835	2

*CVS, cerebral vasospasm; NA, not applicable*.

## Discussion

Nine studies in English were included in our research, and no literature from Chinese databases met the inclusion criteria. All patients were randomly assigned to the experimental and control groups, with the study population ranging from 25 to 803. We defined mRS ≤ 2 or GOS ≥ 4 as favorable prognosis, and a mRS score of 3–6 or GOS of 1–3 as poor prognosis. Our research aimed to evaluate the efficacy and safety of statins in patients with aSAH.

This meta-analysis indicated that a short treatment course of statins over a period of 2 weeks may improve the neurological prognosis and that statins had no favorable effect on neurological prognosis when administered continuously for 21 days. Statins were associated with reduced CVS but they also increased bacteremia.

Statins are the most commonly used lipid-lowering drugs (Lin et al., 2015). Previous studies have indicated that statins increase serum cholesterol clearance by competitively inhibiting endogenous cholesterol synthesis of the rate-limiting enzyme HMG-CoA reductase. Meanwhile, some trials have found that statins have a positive effect on the prognosis of aSAH (McGirt et al., 2002; Ferro et al., 2008).

It has been demonstrated that high lipid levels increase the risk of poor prognosis for aSAH, possibly because high lipid levels promote aneurysm wall inflammation and induce vascular smooth muscle cell death (Lindbohm et al., 2018). In general, vascular smooth muscle cells can repair damaged aneurysm walls and delay the progression of inflammation. Additionally, the effect of statins on prognosis may also be independent of the mechanism of lowering lipid levels (Tada et al., 2011; Rowland et al., 2012; Woo et al., 2015). Statins are known to inhibit the migration and proliferation of white blood cells into the blood vessels, activate cytokines, upregulate the expression and activity of endothelial nitric oxide synthase, improve endothelial reactivity, increase cerebral blood flow, and exert antioxidant effects. It is therefore not surprising that the synergistic effects of multiple mechanisms enhance the prognosis of patients with aSAH (Kotlega et al., 2015; Arati et al., 2019).

Considering the two different treatment courses (14 and 21 days), subgroup analysis indicated that a short-course treatment (14 days) had a positive effect on neurological outcomes. Surprisingly, long-course treatment (21 days) of statins did not improve the prognosis. This finding needs further clarification with a larger sample of RCTs. However, more attention should be paid to the course of statin treatment for aSAH.

In terms of safety, our study showed no statistically significant difference in mortality after statin intervention. Shen et al. suggested that statins were not associated with mortality in patients with aSAH and that this condition might be exacerbated by risk factors other than CVS, such as ischemic cerebral infarction (Shen et al., 2019). Another study suggested that statins might be associated with the risk of hemorrhagic stroke (Shen et al., 2017).

CVS is a common complication in patients with aSAH that occurs 4–14 days after bleeding (Smetana et al., 2020). It can lead to severe cerebral infarction and is the leading cause of disability and death in patients, with an incidence of 50–70% (Weir et al., 1978). Shen et al. suggested that statins might prevent CVS, and our results confirmed this conclusion (Shen et al., 2019). Statins can inhibit inflammatory chemokine expression, leukocyte migration, adhesion, and monocyte/macrophage proliferation and facilitate T-helper 2 cell polarization and regulatory T-cell expansion, leading to an increase in gram-negative bacilli infection. Additionally, statins can also alter the metabolic rate of certain antibiotics (i.e., azole antifungals), indirectly causing an increase in bacteremia (Seo et al., 2015). In our study, statins were found to increase bacteremia, but not the level of transaminase or the severity of epilepsy or rhabdomyolysis. Although the incidence of bacteremia increased, statins improved the overall prognosis. These results indicated that the benefit from a 2-week treatment of statin outweighed the detrimental effects. In a retrospective study, statins were found to be associated with an increased risk of pneumonia and other infections (Magulick et al., 2014). A slight increase in bacteremia was found on day 21 of statin treatment compared with day 14, although the statistical difference was not significant (29 vs. 27.5%, *P* = 0.844).

Statins are hepatotoxic, causing elevated transaminase levels in ~3% of patients with aSAH, but they rarely cause clinical symptoms (Russo et al., 2014; Benes et al., 2016). In our study, elevated transaminase levels in patients were not statistically significant due to the limited sample size. Rhabdomyolysis is a rare complication associated with statins, and its mechanism is still unclear (Simons et al., 2015). Rhabdomyolysis was reported in only two trials included in our study, and the insufficient sample size might be the reason for the lack of significant differences or a strong conclusion in this regard.

Statins differ in their pharmacological characteristics. For example, pravastatin is hydrophilic and has a half-life of 1–3 h, which is significantly shorter than that of atorvastatin and simvastatin. Thus, it hardly crosses the blood-brain barrier. Atorvastatin and simvastatin are lipophilic drugs with significant side effects, including muscle symptoms. Therefore, there might be differences in clinical outcomes among several statins (Sirtori, 2014). In our meta-analysis, only one study focused on epilepsy, and the risk of epilepsy was similar in both the statin and placebo groups (Kirkpatrick et al., 2014). In the included studies using a lower dose of simvastatin (40 mg/day) epilepsy was not observed, while Lin et al. indicated that a high-dose treatment of statins such as atorvastatin and rosuvastatin leads to epilepsy (Lin et al., 2018). Further investigations of epilepsy in patients on statin treatment are warranted in the future.

This meta-analysis had several strengths. First, we searched different databases to identify relevant RCTs. Further, our study provided a comprehensive overview of the efficacy and safety of statin administration in aSAH subjects. Moreover, by analyzing the data of all the included RCTs on poor neurological prognosis, all-cause mortality, and statin-related complications such as CVS, bacteremia, epilepsy, elevated transaminase, and rhabdomyolysis, we further confirmed that the use of statins is related to the incidence of CVS and bacteremia.

Nevertheless, our study has some limitations. Our broad inclusion criteria led to a heterogeneous population in terms of the length of statin treatment, intervention dose, and disease severity, which might have led to potential bias in the evaluation of the efficacy and safety of statins. In addition, the sample size of some studies was small. There might be an overrepresentation of recently published papers by Chen et al. since it represented nearly a fifth of the included population in our meta-analysis (Chen et al., 2020). Furthermore, because different prognostic indicators (mRS and GOS) were used, there was some uncertainty regarding the estimates. Finally, there was high heterogeneity in the integrated analysis of complications due to insufficient monitoring of complications in various studies.

## Conclusions

This meta-analysis indicated that a short treatment course of statins with a duration of 2 weeks may improve neurological prognosis. Statins were associated with reduced CVS. Although statins increased bacteremia, 2 weeks could be the optimal time window for statin treatment in aSAH according to the pathophysiological characteristics of CVS and the evaluation of prognosis.

## Data Availability Statement

The original contributions presented in the study are included in the article/Supplementary Material, further inquiries can be directed to the corresponding author/s.

## Author Contributions

YB had full access to all the data in the study and takes responsibility for the integrity of the data and the accuracy of the data analysis. TL, SZ, XZ, HJ, and YB concept and design. TL, SZ, YB, and QZ acquisition, analysis, interpretation of data, and statistical analysis. TL, SZ, KL, SH, LL, QZ, XS, and YB critical revision of the manuscript for important intellectual content. QZ, XS, and YB supervision. All authors contributed to the article and approved the submitted version.

## Funding

This project was sponsored by Liaoning Provincial Natural Science Foundation (2020-MS-155), China Medical University novel coronavirus pneumonia prevention and control research project (No. 2020-12-11), Scientific Research Foundation for the Returned Overseas Chinese Scholars, State Education Ministry (2013-1792), and National Science Foundation of China (72074104). The researchers are grateful for the support of several organizations.

## Conflict of Interest

The authors declare that the research was conducted in the absence of any commercial or financial relationships that could be construed as a potential conflict of interest.

## Publisher's Note

All claims expressed in this article are solely those of the authors and do not necessarily represent those of their affiliated organizations, or those of the publisher, the editors and the reviewers. Any product that may be evaluated in this article, or claim that may be made by its manufacturer, is not guaranteed or endorsed by the publisher.
