# Supplementary material for: Optimal Course of Statins for Patients With Aneurysmal Subarachnoid Hemorrhage: Is Longer Treatment Better? A Meta-Analysis of Randomized Controlled Trials
Source: Front Neurosci. 2021 Oct 25;15:757505. doi: 10.3389/fnins.2021.757505 (PMC8573116; doi:10.3389/fnins.2021.757505)
Supplement: Supplementary file 1 [file Table_1.DOCX]

**Supplementary Content**

Table S1. Search strategy.

| **S1.1** | **Pubmed search strategy** |
| --- | --- |
| 1 | "Subarachnoid Hemorrhage"[Mesh] |
| 2 | (((((((((((((((((((((((((((SAH (Subarachnoid Hemorrhage)) OR (SAHs (Subarachnoid Hemorrhage)[Title/Abstract])) OR (Hemorrhage, Subarachnoid[Title/Abstract])) OR (Hemorrhages, Subarachnoid[Title/Abstract])) OR (Subarachnoid Hemorrhages[Title/Abstract])) OR (Subarachnoid Hemorrhage, Aneurysmal[Title/Abstract])) OR (Aneurysmal Subarachnoid Hemorrhage[Title/Abstract])) OR (Aneurysmal Subarachnoid Hemorrhages[Title/Abstract])) OR (Hemorrhage, Aneurysmal Subarachnoid[Title/Abstract])) OR (Hemorrhages, Aneurysmal Subarachnoid[Title/Abstract])) OR (Subarachnoid Hemorrhages, Aneurysmal[Title/Abstract])) OR (Hemorrhage, Spontaneous Subarachnoid[Title/Abstract])) OR (Hemorrhages, Spontaneous Subarachnoid[Title/Abstract])) OR (Spontaneous Subarachnoid Hemorrhage[Title/Abstract])) OR (Spontaneous Subarachnoid Hemorrhages[Title/Abstract])) OR (Subarachnoid Hemorrhages, Spontaneous[Title/Abstract])) OR (Perinatal Subarachnoid Hemorrhage[Title/Abstract])) OR (Hemorrhage, Perinatal Subarachnoid[Title/Abstract])) OR (Hemorrhages, Perinatal Subarachnoid[Title/Abstract])) OR (Perinatal Subarachnoid Hemorrhages[Title/Abstract])) OR (Subarachnoid Hemorrhage, Perinatal[Title/Abstract])) OR (Subarachnoid Hemorrhages, Perinatal[Title/Abstract])) OR (Subarachnoid Hemorrhage, Intracranial[Title/Abstract])) OR (Hemorrhage, Intracranial Subarachnoid[Title/Abstract])) OR (Hemorrhages, Intracranial Subarachnoid[Title/Abstract])) OR (Intracranial Subarachnoid Hemorrhage[Title/Abstract])) OR (Intracranial Subarachnoid Hemorrhages[Title/Abstract])) OR (Subarachnoid Hemorrhages, Intracranial[Title/Abstract]) |
| 3 | 1 OR 2 |
| 4 | "Hydroxymethylglutaryl-CoA Reductase Inhibitors"[Mesh] |
| 5 | (((((((((((((((((((((((Hydroxymethylglutaryl CoA Reductase Inhibitors) OR (Inhibitors, Hydroxymethylglutaryl-CoA Reductase)) OR (Reductase Inhibitors, Hydroxymethylglutaryl-CoA)) OR (HMG-CoA Reductase Inhibitor)) OR (HMG CoA Reductase Inhibitor)) OR (Statin)) OR (Statins, HMG-CoA)) OR (HMG-CoA Statins)) OR (Statins, HMG CoA)) OR (Inhibitors, HMG-CoA Reductase)) OR (Inhibitors, HMG CoA Reductase)) OR (Reductase Inhibitors, HMG-CoA)) OR (HMG-CoA Reductase Inhibitors)) OR (HMG CoA Reductase Inhibitors)) OR (Inhibitors, Hydroxymethylglutaryl-Coenzyme A)) OR (Hydroxymethylglutaryl-Coenzyme A Inhibitors)) OR (Inhibitors, Hydroxymethylglutaryl Coenzyme A)) OR (Inhibitors, Hydroxymethylglutaryl-CoA)) OR (Hydroxymethylglutaryl-CoA Inhibitors)) OR (Inhibitors, Hydroxymethylglutaryl CoA)) OR (Hydroxymethylglutaryl-CoA Reductase Inhibitor)) OR (Hydroxymethylglutaryl CoA Reductase Inhibitor)) OR (Reductase Inhibitor, Hydroxymethylglutaryl-CoA)) OR (Statins) |
| 6 | 4 OR 5 |
| **7** | randomized controlled trial[Publication Type] OR randomized[Title/Abstract] OR placebo[Title/Abstract] |
| 8 | 3 AND 6 AND 7 |
| **S1.2** | **Web of science search strategy** |
| #1 | TS=(Subarachnoid Hemorrhage OR SAH (Subarachnoid Hemorrhage) OR SAHs (Subarachnoid Hemorrhage) OR Hemorrhage, Subarachnoid OR Hemorrhages, Subarachnoid OR Subarachnoid Hemorrhages OR Subarachnoid Hemorrhage, Aneurysmal OR Aneurysmal Subarachnoid Hemorrhage  OR Aneurysmal Subarachnoid Hemorrhages OR Hemorrhage, Aneurysmal Subarachnoid OR Hemorrhages, Aneurysmal Subarachnoid OR Subarachnoid Hemorrhages, Aneurysmal OR Subarachnoid Hemorrhage, Spontaneous OR Hemorrhage, Spontaneous Subarachnoid OR Hemorrhages, Spontaneous Subarachnoid OR Spontaneous Subarachnoid Hemorrhage OR Spontaneous Subarachnoid Hemorrhages OR Subarachnoid Hemorrhages, Spontaneous Perinatal Subarachnoid Hemorrhage OR Hemorrhage, Perinatal Subarachnoid OR Hemorrhages, Perinatal Subarachnoid OR Perinatal Subarachnoid Hemorrhages OR Subarachnoid Hemorrhage, Perinatal OR Subarachnoid Hemorrhages, Perinatal OR Subarachnoid Hemorrhage, Intracranial OR Hemorrhage, Intracranial Subarachnoid OR Hemorrhages, Intracranial Subarachnoid OR Intracranial Subarachnoid Hemorrhage OR Intracranial Subarachnoid Hemorrhages OR Subarachnoid Hemorrhages, Intracranial) |
| #2 | TS=(Hydroxymethylglutaryl CoA Reductase Inhibitors OR Inhibitors, Hydroxymethylglutaryl-CoA Reductase OR Reductase Inhibitors, OR Hydroxymethylglutaryl-CoA OR HMG-CoA Reductase Inhibitor OR HMG CoA Reductase Inhibitor OR Statin OR Statins, HMG-CoA OR HMG-CoA Statins OR Statins, HMG CoA OR Inhibitors, HMG-CoA Reductase OR Inhibitors, HMG CoA Reductase OR Reductase Inhibitors, HMG-CoA OR HMG-CoA Reductase Inhibitors OR HMG CoA Reductase Inhibitors OR Inhibitors, Hydroxymethylglutaryl-Coenzyme A OR Hydroxymethylglutaryl-Coenzyme A Inhibitors Inhibitors, Hydroxymethylglutaryl Coenzyme A OR Inhibitors, Hydroxymethylglutaryl-CoA OR Hydroxymethylglutaryl-CoA Inhibitors OR Inhibitors, Hydroxymethylglutaryl CoA OR Hydroxymethylglutaryl-CoA Reductase Inhibitor OR Hydroxymethylglutaryl CoA Reductase Inhibitor OR Reductase Inhibitor, Hydroxymethylglutaryl-CoA OR Statins) |
| #3 | TS=(random* controlled trial OR random* OR placebo) |
| #4 | #3 AND #2 AND #1 |
| **S1.3** | **Embase search strategy** |
| #59 | #31 AND #57 AND #58 |
| #58 | 'random':ab,ti OR 'placebo':ab,ti OR 'double-blind':ab,ti |
| #57 | #32 OR #33 OR #34 OR #35 OR #36 OR #37 OR #38 OR #39 OR #40 OR #41 OR #42 OR #43 OR #44 OR #45 OR #46 OR #47 OR #48 OR #49 OR #50 OR #51 OR #52 OR #53 OR #54 OR #55 OR #56 |
| #56 | 'statins':ab,ti |
| #55 | 'reductase inhibitor, hydroxymethylglutaryl-coa':ab,ti |
| #54 | 'hydroxymethylglutaryl coa reductase inhibitor':ab,ti |
| #53 | 'hydroxymethylglutaryl-coa reductase inhibitor':ab,ti |
| #52 | 'inhibitors, hydroxymethylglutaryl coa':ab,ti |
| #51 | 'hydroxymethylglutaryl-coa inhibitors':ab,ti |
| #50 | 'inhibitors, hydroxymethylglutaryl-coa':ab,ti |
| #49 | 'inhibitors, hydroxymethylglutaryl coenzyme a':ab,ti |
| #48 | 'hydroxymethylglutaryl-coenzyme a inhibitors':ab,ti |
| #47 | 'inhibitors, hydroxymethylglutaryl-coenzyme a':ab,ti |
| #46 | 'hmg coa reductase inhibitors':ab,ti |
| #45 | 'hmg-coa reductase inhibitors':ab,ti |
| #44 | 'reductase inhibitors, hmg-coa':ab,ti |
| #43 | 'inhibitors, hmg coa reductase':ab,ti |
| #42 | 'inhibitors, hmg-coa reductase':ab,ti |
| #41 | 'statins, hmg coa':ab,ti |
| #40 | 'hmg-coa statins':ab,ti |
| #39 | 'statins, hmg-coa':ab,ti |
| #38 | 'statin':ab,ti |
| #37 | 'hmg coa reductase inhibitor':ab,ti |
| #36 | 'hmg-coa reductase inhibitor':ab,ti |
| #36 | 'hmg-coa reductase inhibitor':ab,ti |
| #35 | 'reductase inhibitors, hydroxymethylglutaryl-coa':ab,ti |
| #34 | 'inhibitors, hydroxymethylglutaryl-coa reductase':ab,ti |
| #33 | 'hydroxymethylglutaryl coa reductase inhibitors':ab,ti |
| #32 | 'hydroxymethylglutaryl coenzyme a reductase inhibitor'/exp |
| #31 | #1 OR #2 OR #3 OR #4 OR #5 OR #6 OR #7 OR #8 OR #9 OR #10 OR #11 OR #12 OR #13 OR #14 OR #15 OR #16 OR #17 OR #18 OR #19 OR #20 OR #21 OR #22 OR #23 OR #24 OR #25 OR #26 OR #27 OR #28 OR #29 OR #30 |
| #30 | 'subarachnoid hemorrhages, intracranial':ab,ti |
| #29 | 'intracranial subarachnoid hemorrhages':ab,ti |
| #28 | 'intracranial subarachnoid hemorrhage':ab,ti |
| #27 | 'hemorrhages, intracranial subarachnoid':ab,ti |
| #26 | 'hemorrhage, intracranial subarachnoid':ab,ti |
| #25 | 'subarachnoid hemorrhage, intracranial':ab,ti |
| #24 | 'subarachnoid hemorrhages, perinatal':ab,ti |
| #23 | 'subarachnoid hemorrhage, perinatal':ab,ti |
| #22 | 'perinatal subarachnoid hemorrhages':ab,ti |
| #21 | 'hemorrhages, perinatal subarachnoid':ab,ti |
| #20 | 'hemorrhage, perinatal subarachnoid':ab,ti |
| #19 | 'perinatal subarachnoid hemorrhage':ab,ti |
| #18 | 'subarachnoid hemorrhages, spontaneous':ab,ti |
| #17 | 'spontaneous subarachnoid hemorrhages':ab,ti |
| #16 | 'spontaneous subarachnoid hemorrhage':ab,ti |
| #15 | 'hemorrhages, spontaneous subarachnoid':ab,ti |
| #14 | 'hemorrhage, spontaneous subarachnoid':ab,ti |
| #13 | 'subarachnoid hemorrhage, spontaneous':ab,ti |
| #12 | 'subarachnoid hemorrhages, aneurysmal':ab,ti |
| #11 | 'hemorrhages, aneurysmal subarachnoid':ab,ti |
| #10 | 'hemorrhage, aneurysmal subarachnoid':ab,ti |
| #9 | 'aneurysmal subarachnoid hemorrhages':ab,ti |
| #8 | 'aneurysmal subarachnoid hemorrhage':ab,ti |
| #7 | 'subarachnoid hemorrhage, aneurysmal':ab,ti |
| #6 | 'subarachnoid hemorrhages':ab,ti |
| #5 | 'hemorrhage, subarachnoid':ab,ti |
| #4 | 'hemorrhage, subarachnoid':ab,ti |
| #3 | 'sahs (subarachnoid hemorrhage)':ab,ti |
| #2 | 'sah (subarachnoid hemorrhage)':ab,ti |
| #1 | 'subarachnoid hemorrhage'/exp |
| **S1.4** | **Cochrane search strategy** |
| #1 | MeSH descriptor: [Subarachnoid Hemorrhage] explode all trees |
| #2 | (SAH (Subarachnoid Hemorrhage)):ti,ab,kw OR (SAHs (Subarachnoid Hemorrhage)):ti,ab,kw OR (Hemorrhage, Subarachnoid):ti,ab,kw OR (Hemorrhages, Subarachnoid):ti,ab,kw OR (Subarachnoid Hemorrhages):ti,ab,kw (Word variations have been searched) |
| #3 | (Subarachnoid Hemorrhage, Aneurysmal):ti,ab,kw OR (Aneurysmal Subarachnoid Hemorrhage):ti,ab,kw OR (Aneurysmal Subarachnoid Hemorrhages):ti,ab,kw OR (Hemorrhage, Aneurysmal Subarachnoid):ti,ab,kw OR (Hemorrhages, Aneurysmal Subarachnoid):ti,ab,kw (Word variations have been searched) |
| #4 | (Subarachnoid Hemorrhages, Aneurysmal):ti,ab,kw OR (Subarachnoid Hemorrhage, Spontaneous):ti,ab,kw OR (Hemorrhage, Spontaneous Subarachnoid):ti,ab,kw OR (Hemorrhages, Spontaneous Subarachnoid):ti,ab,kw OR (Spontaneous Subarachnoid Hemorrhage):ti,ab,kw (Word variations have been searched) |
| #5 | (Spontaneous Subarachnoid Hemorrhage):ti,ab,kw OR (Spontaneous Subarachnoid Hemorrhages):ti,ab,kw OR (Subarachnoid Hemorrhages, Spontaneous):ti,ab,kw OR (Perinatal Subarachnoid Hemorrhage):ti,ab,kw OR (Hemorrhage, Perinatal Subarachnoid):ti,ab,kw (Word variations have been searched) |
| #6 | (Hemorrhages, Perinatal Subarachnoid):ti,ab,kw OR (Perinatal Subarachnoid Hemorrhages):ti,ab,kw OR (Subarachnoid Hemorrhage, Perinatal):ti,ab,kw OR (Subarachnoid Hemorrhages, Perinatal):ti,ab,kw OR (Subarachnoid Hemorrhage, Intracranial):ti,ab,kw (Word variations have been searched) |
| #7 | (Hemorrhage, Intracranial Subarachnoid):ti,ab,kw OR (Hemorrhages, Intracranial Subarachnoid):ti,ab,kw OR (Intracranial Subarachnoid Hemorrhage):ti,ab,kw OR (Intracranial Subarachnoid Hemorrhages):ti,ab,kw OR (Subarachnoid Hemorrhages, Intracranial):ti,ab,kw (Word variations have been searched) |
| #8 | #1 OR #2 OR #3 OR #4 OR #5 OR #6 OR #7 |
| #9 | MeSH descriptor: [Hydroxymethylglutaryl-CoA Reductase Inhibitors] explode all trees |
| #10 | (Hydroxymethylglutaryl CoA Reductase Inhibitors):ti,ab,kw OR (Inhibitors, Hydroxymethylglutaryl-CoA Reductase):ti,ab,kw OR (Reductase Inhibitors, Hydroxymethylglutaryl-CoA):ti,ab,kw OR (HMG-CoA Reductase Inhibitor):ti,ab,kw OR (HMG CoA Reductase Inhibitor):ti,ab,kw (Word variations have been searched) |
| #11 | (Statin):ti,ab,kw OR (Statins, HMG-CoA):ti,ab,kw OR (HMG-CoA Statins):ti,ab,kw OR (Statins, HMG CoA):ti,ab,kw OR (Inhibitors, HMG-CoA Reductase):ti,ab,kw (Word variations have been searched) |
| #12 | (Inhibitors, HMG CoA Reductase):ti,ab,kw OR (Reductase Inhibitors, HMG-CoA):ti,ab,kw OR (HMG-CoA Reductase Inhibitors):ti,ab,kw OR (HMG CoA Reductase Inhibitors):ti,ab,kw OR (Inhibitors, Hydroxymethylglutaryl-Coenzyme A):ti,ab,kw (Word variations have been searched) |
| #13 | (Hydroxymethylglutaryl-Coenzyme A Inhibitors):ti,ab,kw OR (Inhibitors, Hydroxymethylglutaryl Coenzyme A):ti,ab,kw OR (Inhibitors, Hydroxymethylglutaryl-CoA):ti,ab,kw OR (Hydroxymethylglutaryl-CoA Inhibitors):ti,ab,kw OR (Inhibitors, Hydroxymethylglutaryl CoA):ti,ab,kw (Word variations have been searched) |
| #14 | (Hydroxymethylglutaryl-CoA Reductase Inhibitor):ti,ab,kw OR (Hydroxymethylglutaryl CoA Reductase Inhibitor):ti,ab,kw OR (Reductase Inhibitor, Hydroxymethylglutaryl-CoA):ti,ab,kw OR (Statins):ti,ab,kw (Word variations have been searched) |
| #15 | #9 OR #10 OR #11 OR #12 OR #13 OR #14 |
| #16 | #8 AND #15 |
